# Supplementary material for: Genome taxonomy of the genus Neptuniibacter and proposal of Neptuniibacter victor sp. nov. isolated from sea cucumber larvae
Source: PLoS One. 2023 Aug 15;18(8):e0290060. doi: 10.1371/journal.pone.0290060 (PMC10426996; doi:10.1371/journal.pone.0290060)
Supplement: S1 Table — PG, phosphatidylglycerol; PE, phosphatidylethanolamine; DPG, diphosphatidylglycerol; PL, phospholipids; AL, aminolipid; PN, phosphoaminolipid; nd: not determined. (PDF) [file pone.0290060.s001.pdf]

S1 Table. Chemotaxonomic profile of previously described *Neptuniibacter*

	Neptuniibacter halophilus	*Neptuniibacter caesariensis*	*Neptuniibacter marinus*	*Neptuniibacter pectenicola*
**Predominant FA**				
iso-C10:0	1.2	nd	nd	nd
anteiso-C11:0	1.8	nd	nd	nd
C14:0	1.1	nd	1.3	tr
C16:0	15.5	16.1	25.7	22.8
C16:1ω7c and/or C16:1ω6c and/or C15 iso 2-OH	39.8	41.2	36.8	23.0
C18:0	1.8	1.0	4.1	1.9
C18:1ω7c and/or C18:1ω6c	29.4	35.9	23.7	37.0
19:0 10-methyl	nd	nd	nd	1.9
C10:0 3OH	7.5	5.0	5.1	8.7
C11:0 2OH	1.6			
C12:1 3OH	nd	nd	1.4	2.0
**Major lipid class**				
PG	+	+	+	+
PE	+	+	+	+
DPG	nd	nd	+	+
PL	PL1 : +<br>PL2 : -	nd	+	+
AL	AL1 : +<br>AL2 : +	nd	+	-
PN	nd	nd	+	+
**Ubiquinone**				
	Q8	Q8	Q8	Q8

PG, phosphatidylglycerol; PE, phosphatidylethanolamine; DPG, diphosphatidylglycerol; PL, phospholipids; AL, aminolipid; PN, phosphoaminolipid; nd: not determined.
