# Supplementary material for: Genome taxonomy of the genus Neptuniibacter and proposal of Neptuniibacter victor sp. nov. isolated from sea cucumber larvae
Source: PLoS One. 2023 Aug 15;18(8):e0290060. doi: 10.1371/journal.pone.0290060 (PMC10426996; doi:10.1371/journal.pone.0290060)
Supplement: S2 Fig — Reference genomes were retrieved from NCBI database. (PDF) [file pone.0290060.s003.pdf]

	N. victor PT1^{T}	*N. caesariensis*^{T}	*N. halophilus*^{T}	*N. marinus*^{T}	*N. pectenicola*^{T}
Neptuniibacter victor PT1^{T}	100.0	75.8	74.8	77.8	77.8
Neptuniibacter caesariensis^{T}	75.8	100.0	76.3	73.5	73.2
Neptuniibacter halophilus^{T}	74.8	76.3	100.0	73.6	73.5
Neptuniibacter marinus^{T}	77.8	73.5	73.6	100.0	87.7
Neptuniibacter pectenicola^{T}	77.8	73.2	73.5	87.7	100.0

S2 Fig. Heat map representation of AAI values.

Reference genomes were retrieved from NCBI database.
