# Supplementary material for: Genome taxonomy of the genus Neptuniibacter and proposal of Neptuniibacter victor sp. nov. isolated from sea cucumber larvae
Source: PLoS One. 2023 Aug 15;18(8):e0290060. doi: 10.1371/journal.pone.0290060 (PMC10426996; doi:10.1371/journal.pone.0290060)
Supplement: S4 Fig — Predicted fatty acid synthetic pathway in Neptuniibacter. ACP: acyl-carrier protein; accABCD: acetyl-CoA carboxylase complex; fabA: 3-hydroxyacyl-ACP dehydrase/trans-2-decanoyl-ACP isomerase; fabB: 3-ketoacyl-ACP synthase Ⅰ; fabD: malonyl-CoA: ACP transacylase; fabF: 3-ketoacyl-ACP synthase Ⅱ; fabG: 3-ketoacyl-ACP reductase; fabI: enoyl-ACP reductase Ⅰ; fabV: enoyl-ACP reductase; fabY: 3-ketoacyl-ACP synthase; fabZ: 3-hydroxyacyl-ACP dehydratase; lpxA: glucosamine N-acyltransferase. (PDF) [file pone.0290060.s005.pdf]

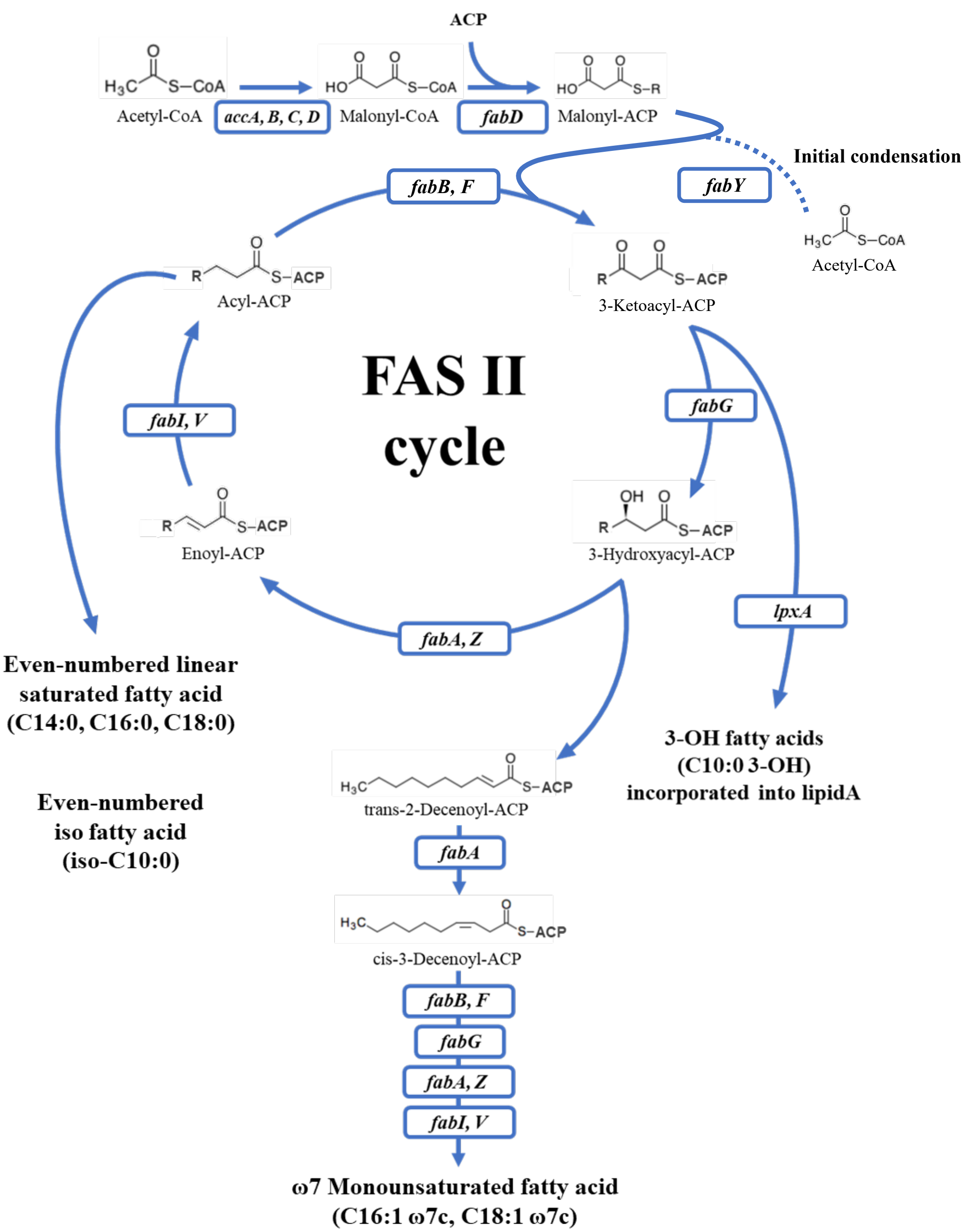

S4 Fig. Predicted fatty acid synthetic pathway in *Neptuniibacter*.

ACP: acyl-carrier protein; *accABCD*: acetyl-CoA carboxylase complex; *fabA*: 3-hydroxyacyl-ACP dehydrase/trans-2-decanoyl-ACP isomerase; *fabB*: 3-ketoacyl-ACP synthase I; *fabD*: malonyl-CoA: ACP transacylase; *fabF*: 3-ketoacyl-ACP synthase II; *fabG*: 3-ketoacyl-ACP reductase; *fabI*: enoyl-ACP reductase I; *fabV*: enoyl-ACP reductase; *fabY*: 3-ketoacyl-ACP synthase; *fabZ*: 3-hydroxyacyl-ACP dehydratase; *lpxA*: glucosamine N-acyltransferase.
