# Supplementary figures and images for: Genome taxonomy of the genus Neptuniibacter and proposal of Neptuniibacter victor sp. nov. isolated from sea cucumber larvae
Source: PLoS One. 2023 Aug 15;18(8):e0290060. doi: 10.1371/journal.pone.0290060 (PMC10426996; doi:10.1371/journal.pone.0290060)

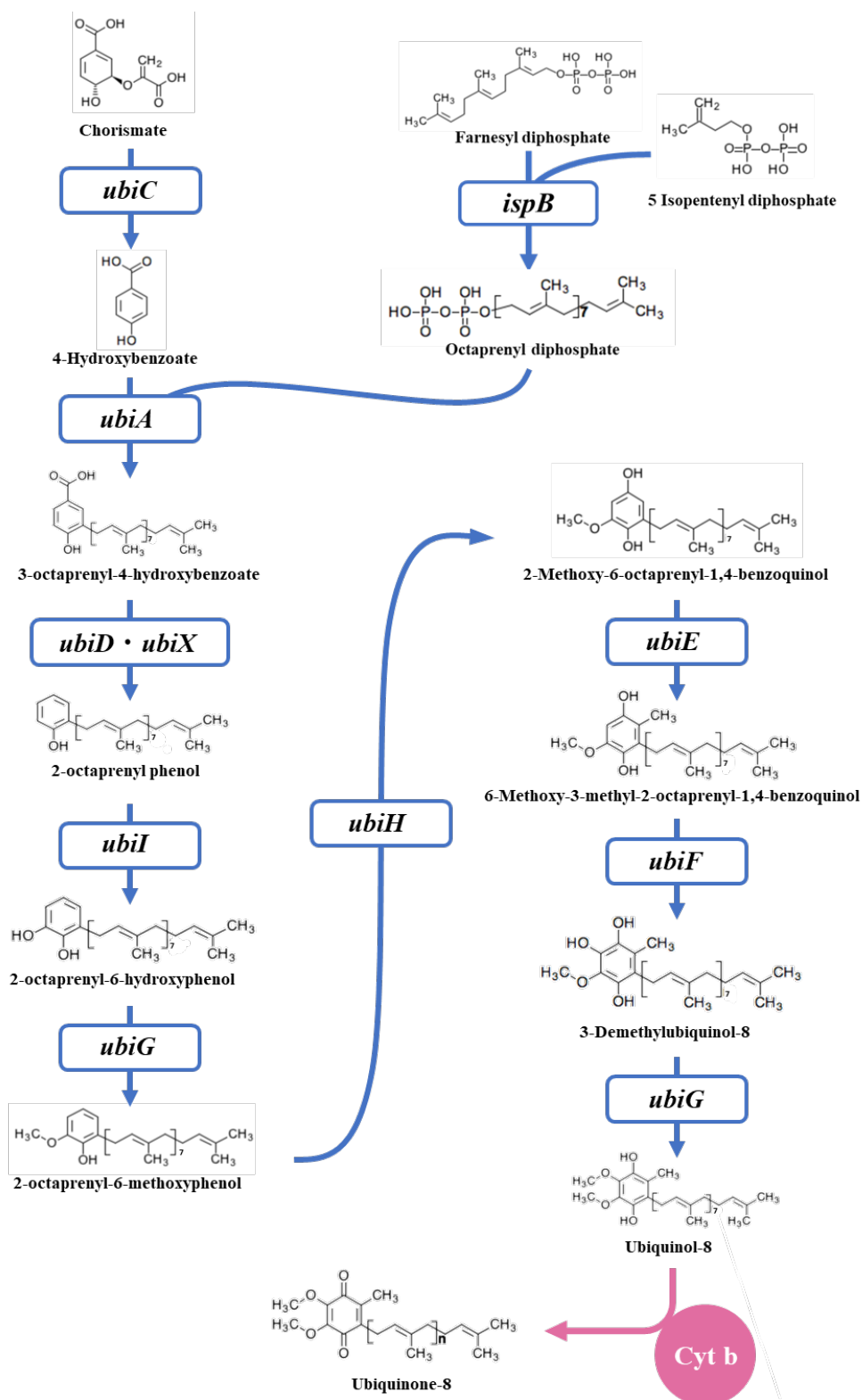

S7 Fig. Predicted Q-8 synthetic pathways in *Neptuniibacter*.

Supplement: S7 Fig — (PDF) [file pone.0290060.s008.pdf]
