# Supplementary material for: Genome taxonomy of the genus Neptuniibacter and proposal of Neptuniibacter victor sp. nov. isolated from sea cucumber larvae
Source: PLoS One. 2023 Aug 15;18(8):e0290060. doi: 10.1371/journal.pone.0290060 (PMC10426996; doi:10.1371/journal.pone.0290060)
Supplement: S3 Fig — 0: negative, 1:phypat positive, 2: phypat+PGL positive, 3: both predictions positive. (PDF) [file pone.0290060.s004.pdf]

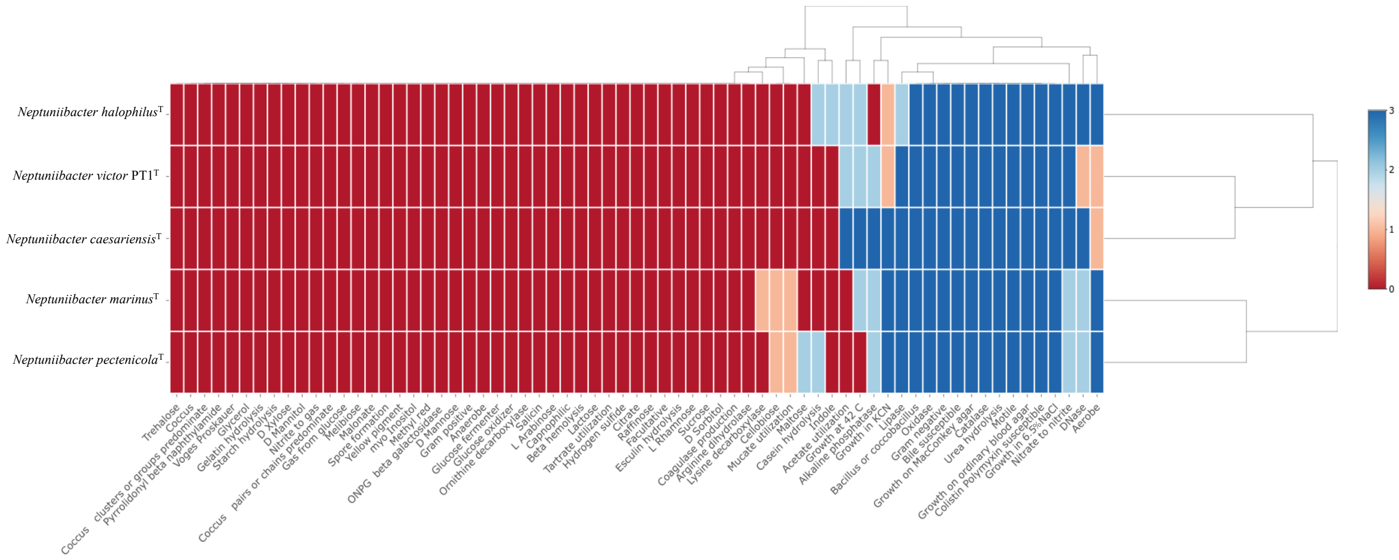

S3 Fig . *In silico* phenotyping of PT1 by Traitar. 0: negative, 1:phypat positive, 2: phypat+PGL positive, 3: both predictions positive.
