# Supplementary material for: Genome taxonomy of the genus Neptuniibacter and proposal of Neptuniibacter victor sp. nov. isolated from sea cucumber larvae
Source: PLoS One. 2023 Aug 15;18(8):e0290060. doi: 10.1371/journal.pone.0290060 (PMC10426996; doi:10.1371/journal.pone.0290060)
Supplement: S6 Fig — Protein/enzyme name each gene is coding: fabA: 3-hydroxyacyl-ACP dehydrase/trans-2-decanoyl-ACP isomerase; fabB: 3-ketoacyl-ACP synthase Ⅰ; fabD: malonyl-CoA: ACP transacylase; fabF: 3-ketoacyl-ACP synthase Ⅱ; fabG: 3-ketoacyl-ACP reductase; fabI: enoyl-ACP reductase Ⅰ; fabV: enoyl-ACP reductase; fabY: 3-ketoacyl-ACP synthase; fabZ: 3-hydroxyacyl-ACP dehydratase; acpP: Acyl-carrier protein; plsX: FA/phospholipid synthesis protein; lpxAD: glucosamine N-acyltransferase; lpxB: lipid-A-disaccharide synthase. (PDF) [file pone.0290060.s007.pdf]

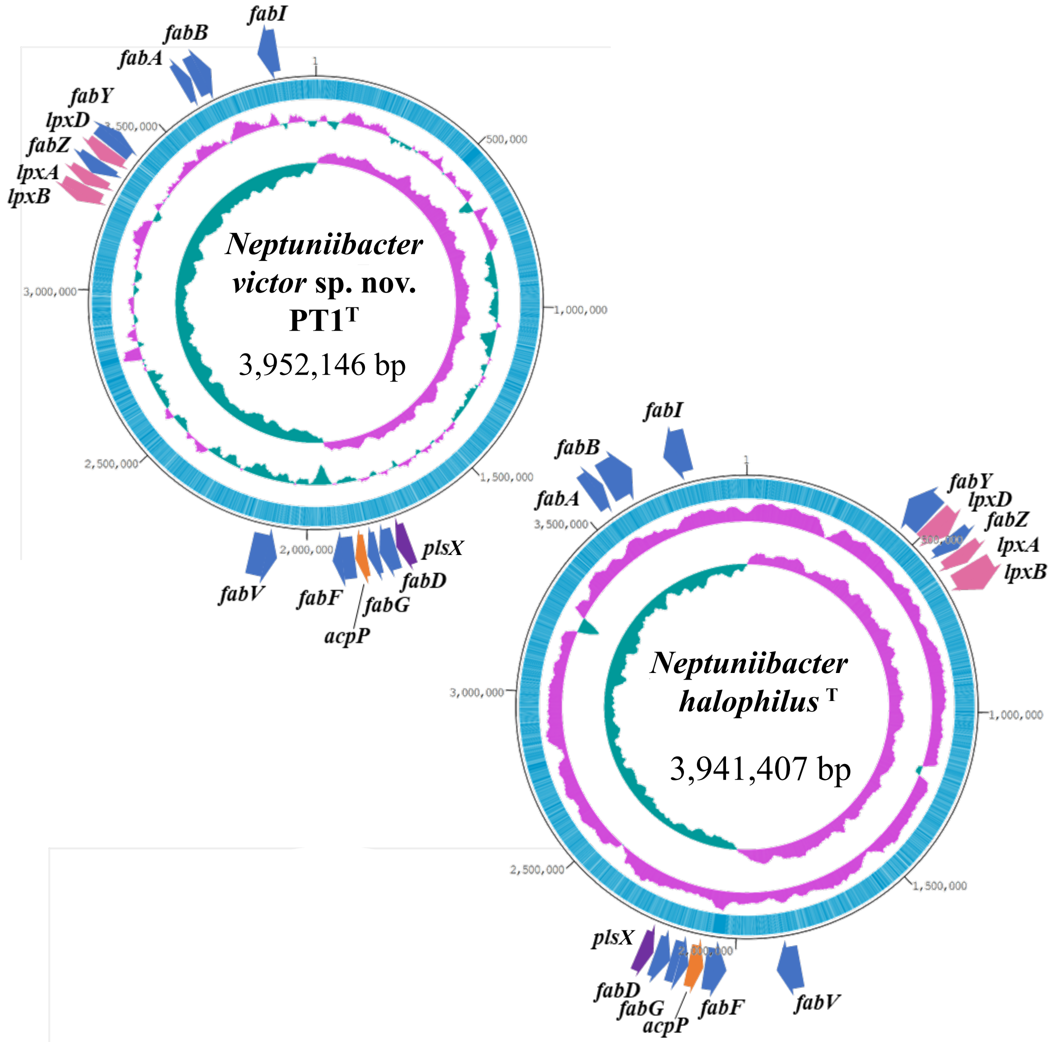

S6 Fig . Genomic distribution of *fab* and associated genes.

Protein/enzyme name each gene is coding: *fabA*: 3-hydroxyacyl-ACP dehydrase/trans-2-decanoyl-ACP isomerase; *fabB*: 3-ketoacyl-ACP synthase I; *fabD*: malonyl-CoA: ACP transacylase; *fabF*: 3-ketoacyl-ACP synthase II; *fabG*: 3-ketoacyl-ACP reductase; *fabI*: enoyl-ACP reductase I; *fabV*: enoyl-ACP reductase; *fabY*: 3-ketoacyl-ACP synthase; *fabZ*: 3-hydroxyacyl-ACP dehydratase; *acpP*: Acyl-carrier protein; *plsX*: FA/phospholipid synthesis protein; *lpxAD*: glucosamine N-acyltransferase; *lpxB*: lipid-A-disaccharide synthase.
