# Supplementary material for: Genome taxonomy of the genus Neptuniibacter and proposal of Neptuniibacter victor sp. nov. isolated from sea cucumber larvae
Source: PLoS One. 2023 Aug 15;18(8):e0290060. doi: 10.1371/journal.pone.0290060 (PMC10426996; doi:10.1371/journal.pone.0290060)
Supplement: Attachment — Submitted filename: 13Mar23_response_DRA.docx [file pone.0290060.s009.docx]

Dear Dr. Sedlar, Academic Editor, PLOS ONE

We gratefully thank to the editor for comments concerning our manuscript entitled “Genome taxonomy of the genus Neptuniibacter and proposal of Neptuniibacter victor sp. nov. isolated from sea cucumber larvae”, PONE-D-23-05318.

We have described accession numbers and these are now publicly available.

Academic Editor: Thank you for submitting your manuscript to PLOS ONE. Before I'll send your manuscript to reviewers, I need to secure that the manuscript meets publication criteria. Since the genome assembly is a part of your manuscript for the sake of reproducibility you need to make all data publicly available. Therefore, you need to upload raw sequencing data to Sequence Read Archive or similar database. Moreover, your accessions for N. halophilus LMG 25378T are not currently available in GenBank/EMBL/DDBJ. Please make all neccessary data available for peer review.

Reply: Thank you for your comments. We provide DRA numbers and all sequences are now publicly available.
