# Supplementary material for: Genome taxonomy of the genus Neptuniibacter and proposal of Neptuniibacter victor sp. nov. isolated from sea cucumber larvae
Source: PLoS One. 2023 Aug 15;18(8):e0290060. doi: 10.1371/journal.pone.0290060 (PMC10426996; doi:10.1371/journal.pone.0290060)
Supplement: Attachment — Submitted filename: Response_letter_revision_1July23.docx [file pone.0290060.s010.docx]

Dear Professor Karel Sedlar, the editor, and reviewers,

We appreciate editor and reviewers for constructive suggestions for PONE-D-23-05318R1. We improved the manuscript according to the editorial office and reviewers’ comments. Responses for specific comments are described as follows. All changes were found in the tracking-on file separately uploaded. We also refine Tables 1 and 2, and Figs 1-3. Table 1 was not framed in body, so we also attached table1.tif as other material.

Editorial office

1. During our internal evaluation of the manuscript, we found significant text overlap between your submission and your previously published work (In silico chemical taxonomy section in particular)

Response: Thank you for the suggestion. We used almost same approach of previously published papers, so many parts of body text were overlapped. However, we did remove redundancy and put citations of previous paper.

Reviewer: 1

1. The article mentions that the Unicycler tool was used for genome assembly. However, it is not clear if any preprocessing was done on the Illumina data, such as adapter removal, which can significantly influence results. Moreover, I am missing information on whether the quality of the sequencing data was checked.

Response: Thank you so much for the comments after careful reading. We did not perform preprocessing Illumina reads at this time because we confirmed QV>27 and no adaptor and barcode sequences of the Illumina read using the FastQC program before the assembly. We put the explanation in body.

2. For pangenome analyses;

1) The author states that whole genome sequences were obtained, and as a result, contigs were obtained (Tab. 1). In the case of more contigs, it is unclear if contigs contain only chromosome sequences or if plasmids are also present in the assembled contigs. If yes, are genes from plasmids included in the pan-genome analysis?

2) In the section on pan and core genome analysis, it is not clear if the same tool was used for both analyses.

3) In the section Pan and core genomes and its ecogenomic interpretation, the author mentions that the assimilatory nitrate reduction pathway was predicted in the analyzed strain. It is unclear how this pathway was predicted and if a specific tool was used.

Response:

1) Thank you so much for the comments. Actually, pan-genome analyses only use genomes, but unfortunately, we did not obtain complete genome sequences ourselves from the other three *Neptuniibacter* type strains. Due to being not survived during shipment from European collections by the heat wave in Europe in 2022 (too hot summer during shipments), we were unable to obtain those type strains. So, we decided to use draft genomes obtained from public databases. These sequences might include plasmids, but we could not select and differentiate them. PT1 has no plasmid, but *N. halophilus* has one plasmid.

2) We used Anvio for both pan- and core- analyses. We modified body in related parts.

3) For assimilatory nitrate reduction, we evaluated only by presence of *nasAC* and *nirA* genes unique in PT1. We added one more reference for that.

3. For the other methodology

1) On line 107, the article states that the ML tree was generated from 100 replicates, but Figure 1 indicates that 1000 replicates were performed. This discrepancy should be clarified.

2) The article references the acceptance of results according to formula 2 for the OGRIs calculation (line 115), but formula 2 is not provided.

3) While AAI values were calculated for PT1T and other *Neptuniibacter* species, the obtained values are not provided.

4) It is unclear how the author determined which genomes were used or not for analysis, as indicated in Table 1. The same issue applies to the MLSA column in Table 1.

5) The article states that phylogenetic network reconstruction was performed for MLSA, but there is no description of the settings used for the SplitsTree tool or the method of construction used.

Response: Thank you for your comments. All points were fixed and/or corrected accordingly. Rerated to 1) and 5), we refined 16S and MLSA network as well, in particular, regions used for those phylogenetic analyses were described. For 2), we modified the body to cover formula 2 explanation by ref 17, in which it is explained that Formula 2 is optimized for using incomplete genomes. We both analyzed complete and incomplete genomes, so we selected formula 2. AAI values were already provided in body around L170 and S2 Fig, but improved the body. To be clear which genome we determined ourselves, we put “in this study” in Table 1. For MLSA, “concatenation of sequences and phylogenetic NeighborNet reconstruction were performed using SplitsTree 4.16.2 with options of Jukes-Cantor correction, gap-exclusion and 1,000 bootstrap”, but eliminating redundancy of previously published paper (Yamano et al., 2023, PLoS One), we add this ref.

4. There are some minor comments regarding the article that I would like to make:

1) The version number should be included for each tool used in this study (e.g. lines 74, 113, 148 etc.).

2) The citation for the MEGAX tool is missing (line 105).

3) The word "Illumina" should start with a capital letter (line 91).

4) In Tab. 1, there should be some units for the size column.

5) Citations need to be sorted in ascending order (line 172).

6) The abbreviation OF is used, but its meaning is not explained (line 72).

Response: Thank you for your comments. All points were fixed and/or corrected accordingly.

Reviewer: 2

1. The authors present a study: Genome taxonomy of the genus *Neptuniibacter* and a proposal of *Neptuniibacter* victor sp. nov. isolated from sea cucumber larvae. The methods used demonstrably confirm that the described strain PT1T belongs to a new, previously undescribed *Neptuniibacter* species. The article is supported by sufficient literature references. The sequencing methods, genome analysis and MLST analysis are described and performed in detail and provide valuable information. On the other hand, my main complaint is that the article provides chemotaxonomic results and phenotyping mainly from the in silico approach. It brings mainly predicted phenotypic characteristics of the studied strain PT1T. I miss in the discussion any mention of the reliability of the analysis with Traitar (Microbial Trait Analyzer) and the used in silico chemical taxonomy and maybe comparison with in vitro methods. In my opinion, a description of genomic and phenotypic traits (tested in vitro) should be more balanced.

2. Other comments and recommendations:

1) Line 127, Table 1: It would be useful to adjust the width of the columns to make the data more readable.

2) Line 185, Table 2: The numbers of the reference type strains should be indicated.

Response: Corrected accordingly.

3) Table 2: The range of the temperatures tested is too wide (4°C, 10°C, ...37°C). It seems reasonable and logical to test the growth also at 20 or 25°C.

Response: We tested 4, 10, 15, 20, 25, 30, 37°C. As indicated in footnote, all strains grow at 15-30°C.

4) The phenotyping data are rather insufficient for the description of the novel species. I suggest testing the commercial kit API ZYM to complement the phenotypic description. This commercial kit is well standardised and has been used in a species description of other *Neptuniibacter* spp. Also test a hydrolysis of gelatine and Tween 80 if possible (tests mentioned in previous *Neptuniibacter* species).

Response: According to the comments, we did perform API20NE, but not APIzym due to no distribution currently in Japan. Tween 80 test was also performed, and described the negative results in body. Traitar prediction results were removed from the description section.

5) Line 187: Please complete the following sentence, there is something missing in the text: Phenotypic data for Neptuniibacter .... are from this study and .... [1-3].

Response: Corrected accordingly.

6) Line 207: Please complete the following sentence, there is something missing in the text: Fig 3. Anvi'o representation of the pangenome of strain PT1T and *Neptuniibacter* ... The word Unique, written in italics, is somewhat confusing together with the species name in Fig. 3. It could be written in brackets.

Response: Changed accordingly.

7) Please include the reference strain numbers in all tables and figures; both in the main text and in the supplementary data.

8) Line 310: There is a mistake in the doi number. The correct one is doi:310 10.1099/ijs.0.64524-0.

Response: Changed accordingly.

9) Please add to the discussion section information on the reliability/confidence of the in silico approaches and assess their suitability for the genera studied.

It would be interesting to compare the predicted properties (Fig. S3) with in vitro tests from previous descriptions of *Neptuniibacter* spp. Do the results agree? In which cases is the gene not expressed?

Response: Thank you so much for the comments. We did compared results by Traitar and experiments, and 82% accuracy in average was evaluated. We did add some results and sentences in related section. We also attached this result here. Finally, we did observe some growth in API20NE system, we did remove the Traitar prediction results from the description.

Fig S7: There is an extra dot.

Response: Changed accordingly.
