# Supplementary material for: Genome taxonomy of the genus Neptuniibacter and proposal of Neptuniibacter victor sp. nov. isolated from sea cucumber larvae
Source: PLoS One. 2023 Aug 15;18(8):e0290060. doi: 10.1371/journal.pone.0290060 (PMC10426996; doi:10.1371/journal.pone.0290060)
Supplement: Attachment — Submitted filename: Response_letter_3rd_revision_25July23.docx [file pone.0290060.s011.docx]

Dear Professor Karel Sedlar, the editor, and reviewers,

We appreciate editor and reviewers for constructive suggestions for PONE-D-23-05318R2. We improved the manuscript according to the editorial office and reviewers’ comments. Responses for specific comments are described as follows. All changes were found in the tracking-on file separately uploaded. Again, Table 1 was not framed in body, so we also attached table1.tif as other material.

Editorial office

1. Please address the text overlap concern we identified between your submission and your previously published work (In silico chemical taxonomy in the Result section in particular):

<https://journals.plos.org/plosone/article?id=10.1371%2Fjournal.pone.0271174>. We would like to make you aware that copying extracts from previous publications, especially outside the methods section, word-for-word is unacceptable. In addition, the reproduction of text from published reports has implications for the copyright that may apply to the publications. Please revise the manuscript to rephrase the duplicated text and fully cite all your sources, where appropriate.

We will carefully review your manuscript upon resubmission, so please ensure that your revision is thorough.

Response: Thank you for the suggestion. Once again, we used almost same approach of previously published papers, so many parts of body text were overlapped. However, we did rewrite, remove redundancy, and add reference citations. Reference format was corrected accordingly.

Reviewer: 1

1. For molecular phylogenetic analysis:

The authors added information about the settings used for MLSA (line 125). However, on line 131, they mentioned that "almost same setting" was used. I am missing information on how the setting was modified.

Response: Thank you so much for the comments. Related part was improved, in particular, line 125 is fused to the first sentence of this section in removing redundancy.

Other comments:

1) Line 28: There is an unnecessary gap between the range of numbers (72.8%- 74.8%).

2) Line 70: In the reference to citations, there are 2 hyphens instead of one.

3) Line 94: I guess QV means quality value, but in the text, it is written as quality vale. The word "Illumina" is missing the letter "m".

4) Line 98: There should be "assembly" instead of "assemble".

5) Line 120: The shortcut DDH is not explained.

6) Line 142: In the word "N. 10aesariensis" the number 10 should be removed.

7) Line 174: There should be "were" instead of "was".

8) Regarding the tools, the referencing of versions should be standardized. Sometimes it is written as "ver.," and other times as "v."

Response: Thank you for the suggestion. All corrected accordingly.
